# Association of a Community Population and Clinic Education Intervention Program With Guideline-Based Aspirin Use for Primary Prevention of Cardiovascular Disease: A Nonrandomized Controlled Trial

**DOI:** 10.1001/jamanetworkopen.2022.11107

**Published:** 2022-05-10

**Authors:** Russell V. Luepker, Milton Eder, John R. Finnegan, Jeremy R. Van’t Hof, Niki Oldenburg, Sue Duval

**Affiliations:** 1 Division of Epidemiology and Community Health, School of Public Health, University of Minnesota, Minneapolis; 2 Cardiovascular Division and Lillehei Heart Institute, University of Minnesota Medical School, Minneapolis; 3 Department of Family Medicine and Community Health, University of Minnesota Medical School, Minneapolis

## Abstract

**Importance:**

Low-dose aspirin is used for primary prevention of cardiovascular disease in approximately one-third of the US adult population. Overuse and underuse are common and not concordant with guidelines.

**Objective:**

To test a community and clinic education intervention to improve guideline-based aspirin use for the primary prevention of cardiovascular disease.

**Design, Setting, and Participants:**

The Ask About Aspirin project was a nonrandomized controlled trial conducted from, July 1, 2015, to March 31, 2020, using professional education, traditional media, and digital media to improve guideline-based aspirin use. The adult population (aged 45-79 years for men and 55-79 years for women) and primary care clinics in Minnesota were the education targets. The 4 adjacent states were controls.

**Interventions:**

The statewide campaign distributed billboards, newspaper articles and other print material, and radio announcements. An Ask About Aspirin website was heavily promoted. Primary care clinics identified appropriate aspirin candidates, and clinicians received continuing education about aspirin.

**Main Outcomes and Measures:**

Guideline-based aspirin use by the target population.

**Results:**

Cross-sectional random telephone surveys of 8342 men aged 45 to 79 years and women aged 55 to 79 years were conducted at baseline, 2 years, and 4 years after the intervention. Participation was similar between men and women (baseline: 973 [49%] vs 1001 [51%]; year 4: 912 [50%] vs 930 [50%]). Age during the study also was similar (baseline: 64.7 [IQR, 64.4-65.1] years; year 4: 66.2 [IQR, 65.8-66.5] years). A validated questionnaire evaluated aspirin use. The Ask About Aspirin website had more than 1 million visits; 124 primary care clinics with more than 1000 participating clinicians were part of the education program. Small, nonsignificant increases in discussions with clinicians regarding aspirin resulted (baseline: 341 of 1001 [34%]; year 4: 339 of 930 [36%]; *P* = .27). Overall aspirin use decreased after the release of new US Preventive Services Task Force guidelines in 2016 and 3 aspirin randomized clinical trials in 2018 suggested reduced aspirin use (baseline: 816 of 1974 [41%]; year 4: 629 of 1842 [34%]; *P* < .001). Decreases were also noted from year 2 to year 4 in appropriate use (year 2: 597 of 1208 [49%]; year 4: 478 of 1191 [40%]; *P* < .001) and overuse (year 2: 170 of 602 [28%]; year 4: 151 of 651 [23%]; *P* = .04). There were no significant differences between Minnesota and the control states.

**Conclusions and Relevance:**

In this nonrandomized controlled trial, a multiyear statewide campaign was not associated with increased appropriate aspirin use for cardiovascular disease prevention. Contextual factors during the project, including guideline changes and media controversy following the new trials, undermined study goals. These findings suggest that although education programs using social media for cardiovascular disease prevention can result in millions of hits, the use of this strategy to encourage behavior change is problematic, even with supportive clinical sites.

**Trial Registration:**

ClinicalTrials.gov Identifier: NCT02607917

## Key Points

**Question:**

Is use of a statewide media and clinician education program associated with improved guideline-based aspirin use for primary prevention of cardiovascular disease?

**Findings:**

In this nonrandomized controlled trial including 8342 adults aged 45 to 79 years in Minnesota, the intervention included traditional and digital media directed to the public plus education for clinicians; Minnesota was compared with surrounding states. Overall aspirin use decreased after release of new guidelines and 3 aspirin trials, and appropriate aspirin use and overuse also decreased; findings in the control group did not differ significantly.

**Meaning:**

In this nonrandomized controlled trial, use of media and clinic education campaigns was not associated with improved guideline-based aspirin use.

## Teaser

This nonrandomized controlled trial examines the association of a multimedia communication with the public and education for health care professionals with guideline-based use of aspirin for prevention of cardiovascular disease (CVD).

## Introduction

Cardiovascular diseases (CVD) remain the leading chronic diseases in the US.^1^ They continue to be a major public health concern despite advances establishing modifiable risk factors and prevention strategies through population, behavioral, and clinic-based actions.^2^ There is a long history of community-based interventions to reduce CVD risk including improved hypertension control, physical activity, better nutrition, and reduced cigarette smoking.^3,4,5,6,7,8^ There have been successes, mixed results, and failures in these programs. Many programs, both current and past, have relied on clinic-based approaches to educate individual patients and traditional media to raise awareness and knowledge of CVD. More recently, digital media health interventions have become more common, especially through social media as the public shifted to online sources of information.^4^ However, less is understood about the potential of these digital strategies for health improvement in a much noisier, ubiquitous, and fragmented electronic media environment.^9,10^

Use of low-dose aspirin is one modifiable factor for the primary prevention of cardiovascular disease recommended for specific or defined subgroups in multiple prevention guideline reports.^11,12,13^ Aspirin is extensively advertised and easily available over the counter at minimal cost. The result is that aspirin is widely used in the adult population with substantial overuse and underuse compared with published guidelines.^14,15,16,17,18,19,20^

The Ask About Aspirin project was initiated in Minnesota in 2015 to increase the appropriate use of aspirin for the primary prevention of myocardial infarction and stroke based on then current guidelines.^12^ The intervention had a 2-prong approach: a statewide aspirin analog and digital media campaign emphasizing a web-based social media strategy, and a health system quality-improvement program in Minnesota clinics to improve guideline adherence. Four surrounding Upper Midwest states functioned as reference controls.

## Methods

The Ask About Aspirin project was a community-based study to reach age-appropriate adults in Minnesota with the surrounding states of Iowa, North Dakota, South Dakota, and Wisconsin serving as reference controls. The intervention spanned July 1, 2015, to December 31, 2019, and the study was completed March 31, 2020. The primary outcome was improvement in appropriate use or nonuse of aspirin according to the 2009 US Preventive Service Task Force (USPSTF) guidelines.^12^ Methods used in this study are briefly described herein and elsewhere.^21^ This study followed the Transparent Reporting of Evaluations With Nonrandomized Designs (TREND) reporting guideline.

### Population

The study population included men aged 45 to 79 years and women aged 55 to 79 years. The survey included items on demographic characteristics (sex, age, marital status, race and ethnicity, and educational level), CVD risk factors, and aspirin contraindications. Race and ethnicity data were collected by National Institutes of Health guidelines, but the numbers are too small for analysis. Because of other ongoing aspirin programs and the challenges in implementing urban clinic-based interventions, study implementation excluded the Minneapolis-St Paul metropolitan area and Rochester populations.

### Safety Assessment

An external data and safety monitoring board was established to evaluate the program’s safety. Senior investigators oversaw every aspect of the program. The protocol was approved by the institutional review board of the University of Minnesota and is available as Supplement 1. Telephone survey participants and health professionals gave verbal or web-based consent for participation. Participants did not receive financial compensation.

### Intervention

The Ask About Aspirin project developed 2 main educational components. The first aimed at increasing public awareness of primary myocardial infarction and stroke prevention using analog and digital media strategies and public relations outlets. The second component aimed to integrate the 2009 USPSTF recommendations into the standard of care throughout the Minnesota health systems. Combining public awareness and individual clinical decision-making suggests a systems approach, exploring the meanings of messages within a context or series of contexts. The Bronfenbrenner ecological or bioecological approach outlines increasingly more abstract meaning and context structures, beginning with the individual (microsystem) and moving into messages and context within which individuals directly (mesosystem) or indirectly (exosystem) interact.^22,23^ In addition, the application of age-specific guidelines points to the further recognition that cognitive development is not purely a matter of brain development in becoming an adult.

### Media Campaign

Study messages within the campaign combined digital and analog media and social media strategies to promote appropriate aspirin use for primary prevention of CVD. Campaign messaging emphasized personal interaction with health care professionals to ensure appropriate use. The media intervention used a professional communication firm experienced in health and public service campaigns (Russell Herder). Media communications were organized as a statewide campaign delivering aspirin messages to an age-appropriate group of individuals in Minnesota through a variety of awareness-building analog media, including print and radio. The strategy integrated well-established media/public relations and advertising tactics, providing a mix of television news coverage, public service announcements, and targeted display messaging. The goal was to draw attention to the preventive use of aspirin and increase general interest and conversation around cardiovascular health.

Digital media platforms provide an individualized, dynamic, and interactive service environment to support behavior change. The campaign-developed Ask About Aspirin website offered cardiovascular health information and key messages developed in tandem with the larger campaign with links to news articles and aspirin-related publications; these activities provided opportunities for individuals to engage with others around messaging content (ie, mesosystem). The media strategy also used search engine optimization techniques to direct traffic to the website, including links from highly visited sites in Minnesota intervention areas and direct links from participating clinics and health care professionals. Analysis of the television, radio, print advertising, and digital strategies estimated more than 24 million exposures statewide per campaign year with an estimated cost per 1000 impressions of $20.83.

In addition to offering information and educational resources, the website included an aspirin candidacy tool. Individuals identified as potential aspirin candidates were advised to discuss a preventive aspirin regimen with their physician. The project’s overall media strategy could not be entirely isolated from adjacent reference states despite principal targeting of age-appropriate adults in Minnesota.

### Health System and Health Professional Intervention

Investigators provided clinical decision tools and resources to support clinic workflow processes optimizing patient aspirin candidacy identification and appropriate aspirin use recommendations. Central to the health professional intervention was a continuing medical education–certified webinar to provide up-to-date preventive aspirin use information to primary care physicians, advanced practice clinicians, and nursing staff. The web-based program equated to 1.0 American Medical Association/Physician Recognition Award category credit for clinicians. These and other materials were updated when the 2016 guidelines were released (eFigure 1 and eFigure 2 in Supplement 2).

Participating clinics identified eligible primary prevention aspirin candidates using the electronic health record*. International Classification of Diseases* and *Current Procedural Terminology* codes excluded patients with atherosclerotic CVD including myocardial infarction, stroke, peripheral artery disease, and revascularization procedures, as well as those with contraindications to aspirin use, such as gastrointestinal bleeding, anticoagulant use, and aspirin allergy. Clinical assessments and discussions related to incorporating aspirin into an individual care plan situate the individual within a social context that is most directly examined in a different ecological systems approach. The exploration by Vygotsky^24^ of how the meaning of messages is dependent on social and cultural contexts includes the concept zone of proximal development, in which change within the individuals is the product of externalized assistance. For our project this concept involves both the primary care clinician and staff (mesosystem) and public health messaging (mesosystem and exosystem).

A recent method that maps self-reported risk factors to the American College of Cardiology/American Heart Association Pooled Cohort Equations for 10-year CVD risk was used to establish risk status.^25^ Many clinics integrated the patient eligibility criteria into an electronic medical record best-practice alert, assisting clinicians to identify patients for whom aspirin was appropriate or not appropriate.

Each participating clinic identified a staff lead to facilitate communication. These individuals typically functioned as clinic champions. A study practice facilitator contacted each clinic to introduce project resources available including printed materials. Practice facilitators met with clinic leaders and teams quarterly, talking in-person or through video conferences. This process is detailed elsewhere.^26^

### Outcome: Aspirin Use for Primary Prevention

A cross-sectional survey of noninstitutionalized resident adults (men aged 45-79 years and women aged 55-79 years) was conducted at baseline and in project years 2 and 4. For each survey, households with land-line phones were used to generate a random sample of 2400 age- and sex-targeted adults from Minnesota. A random sample of 1200 age- and sex-targeted adults was simultaneously collected in the 4 Upper Midwest control states. Following a presurvey study invitation via a mailed letter, a 10-minute telephone survey was administered by trained interviewers asking about CVD history and risk, aspirin use, aspirin-related physician discussions, and media exposure to aspirin-related content (eAppendix in Supplement 2). Self-reported aspirin use (every day or every other day) or nonuse where appropriately indicated was the primary outcome. Individuals who used aspirin for secondary CVD prevention were excluded. In a previously published report using the same telephone survey, the validity of self-reported aspirin use was tested with blood levels of thromboxane B_2_, an indicator of aspirin use.^27^ Sensitivity and specificity of this questionnaire were more than 90%, supporting the validity of the telephone survey for aspirin use. The validity of self-reported telephone history of CVD risk factors, such as hypertension and blood cholesterol levels, was tested against measured values in a population survey. A high concordance was found between this interview and the actual risk factor measurement.^25^

### Statistical Analysis

Data are presented as number (percent) for categorical variables and mean (SD) for continuous variables. For comparisons of aspirin use between time points, χ^2^ tests were used. All statistics were weighted by the source target population size; thus, percentages may not add to 100% or be calculated directly from numerators and denominators.

We considered the survey year as a continuous variable in linear and logistic regression models to examine the statistical significance of trends over time. Similarly, these models were used to compare trends across surveys between Minnesota and the control states.

All tests were 2-sided, and *P* < .05 was deemed to be statistically significant. Analyses were performed in Stata, version 16.1 (StataCorp LLC).

## Results

The number of participants in the population surveys are reported in eTable 1 in Supplement 2. A total of 5626 eligible adults were surveyed in 3 waves in Minnesota. In the surrounding states, 2716 individuals were surveyed over the same period. The participation rate for this telephone survey averaged 49%. By design, men and women were sampled equally (baseline: 973 [49%] vs 1001 [51%]; year 2: 914 [50%] vs 896 [50%]; year 4: 912 [50%] vs 930 [50%]). The demographic and health characteristics of the populations appear in eTable 2 in Supplement 2. Between the baseline and year 4 surveys, the mean age increased somewhat (baseline: 64.7 [IQR, 64.4-65.1] years; year 2: 65.6 [IQR, 65.3-66.0] years; year 4: 66.2 [IQR, 65.8-66.5] years). The self-reported race of the population was mainly White (97.5%) vs other races and ethnicities (African American, 0.07%; Asian, 0.07%; Native American, 0.68%; Pacific Islander, 0.07%; multiracial, 0.66%; and unknown, 1.05%), which was representative of the surveyed areas.^28^ Risk factors for CVD, including hypertension, hyperlipidemia, diabetes, and smoking, were similar over the 3 surveys, after adjustment for age. Aspirin contraindications, including gastrointestinal bleeding, allergy, and anticoagulant use, were also similar. Baseline measures did not differ substantially between Minnesota and the control states (eTable 2 in Supplement 2).

### Intervention

#### Analog Media

The program used analog media to reach the population. For the initial 6 months (May-October 2015), billboards were placed at 58 locations throughout Minnesota (eFigure 3 in Supplement 2). Most were changed at 3 months, but some stayed additional months. In addition, radio announcements were distributed to local stations along with articles to local newspapers and content for health system newsletters during the study.

#### Digital Media

The Ask About Aspirin website was promoted through paid placements on Google, Yahoo, Facebook, Pandora, Twitter, and other social media channels. Because most responses were received through these channels, Google, Yahoo and Facebook were maintained throughout the study. The Figure shows the unique and total visits to the website. The number of visits increased steadily over the years of the study with total visits rising to more than 1 million. Of the 627 334 unique visitors, 70 859 entered the assessment website, with 53 517 completing the self-assessment for aspirin candidacy. Of these visitors, 28 038 (52%) completed the appropriate use questions and were advised to seek further advice from their physician as potential aspirin candidates. Another 25 479 (48%) assessment completers were considered not to be aspirin candidates. Because of privacy considerations, there are no data specifically linking the advice offered through the website and individual follow-up.

**Figure.**
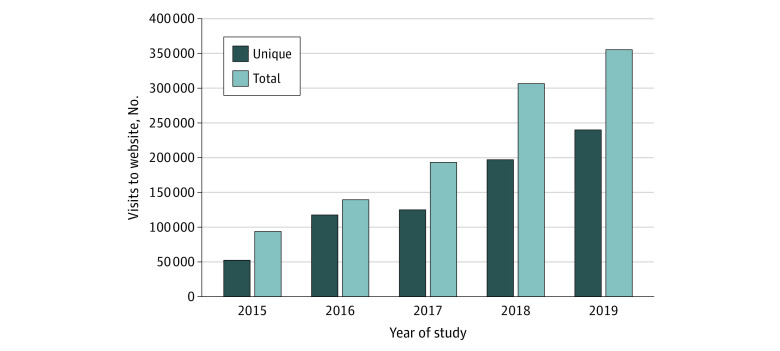
Unique and Total Visits to the Ask About Aspirin Website

#### Health System and Health Professional Intervention

A total of 15 of the 68 (22%) largest Minnesota health systems with primary care clinics participated in the Ask About Aspirin project. This included 124 clinics or 36% of the total primary care clinics among all the health systems.

The continuing medical education webinar was delivered to participating clinics in group settings or to individuals through the internet. Physicians from participating clinics completing the 1-credit continuing medical education module numbered 378 (51%) of 744 total physicians. In addition, 747 of 1306 clinic medical staff participated (57%), including physician assistants, nurse practitioners, nurses, and other professionals with direct patient contact.

#### Aspirin Use and Related Behaviors

Reported awareness of the Ask About Aspirin media campaign in Minnesota ranged from 4% to 6% (Table 1) and did not show a time trend. The surrounding states had lower levels of awareness throughout the study period (2%-3%).

**Table 1.** Primary Prevention Participants Who Saw or Heard About Ask About Aspirin: 2015-2020

Variable	No./No. (%)		
	Baseline	Year 2	Year 4
**Minnesota**			
Women	59/1001 (6)	50/896 (6)	48/930 (5)
Men	46/973 (5)	51/914 (6)	39/912 (4)
**Control states**			
Women	15/492 (3)	15/456 (3)	9/466 (2)
Men	14/481 (3)	8/362 (2)	12/459 (3)

Table 2 displays results of messages heard that favored or did not favor aspirin use. At baseline and the year 2 survey, the messages were predominantly favorable but did not differ significantly between Minnesota and the control states. However, in year 4, the number of messages not in favor of aspirin use for primary prevention of CVD increased significantly and were more often seen or heard in Minnesota. Participants reporting mixed messages (ie, both positive and negative) increased sharply between the year 2 and year 4 surveys in all states.

**Table 2.** Responses to Messages About Use of Aspirin for Primary Prevention of MI or Stroke: 2015-2020^a^

Variable	No./No. (%)	
	Minnesota	Control states
**Baseline**		
Saw or heard messages	1291/1974 (65)	677/973 (70)
In favor^b^	1059/1291 (82)	547/677 (81)
Not in favor	69/1291 (5)	34/677 (5)
Don’t know	163/1291 (13)	96/677 (14)
Did not see or hear messages	683/1974 (35)	296/973 (30)
**Year 2**		
Saw or heard messages	1105/1810 (61)	511/818 (62)
In favor^c^	998/1105 (90)	453/511 (89)
Not in favor	44/1105 (4)	28/511 (5)
Don’t know	63/1105 (6)	30/511 (6)
Did not see or hear messages	705/1810 (39)	307/818 (38)
**Year 4**		
Saw or heard messages	1244/1842 (68)	592/925 (64)
In favor^d^	825/1244 (66)	413/592 (70)
Not in favor	363/1244 (29)	161/592 (27)
Don’t know	56/1244 (5)	18/592 (3)
Did not see or hear messages	598/1842 (32)	333/925 (36)

Abbreviation: MI, myocardial infarction.

^a^ Survey questions related to these results (QF4 and QF5) are given in the eAppendix in the Supplement.

^b^ *P* = .61 for Minnesota vs control states.

^c^ *P* = .30 for Minnesota vs control states and *P* < .001 for 2-year vs 4-year differences for both Minnesota and control states.

^d^ *P* = .14 for Minnesota vs control states.

Trends in aspirin use discussions with clinicians are reported in Table 3. In Minnesota, patient and health care professional–initiated discussions increased, but nonsignificantly (baseline: 341 of 1001 [34%]; year 4: 339 of 930 [36%]; *P* = .27). Trends in discussions were more variable in the control states. The trends in aspirin use discussions did not differ significantly between Minnesota and the control states, although the level of discussion was uniformly higher in Minnesota.

**Table 3.** Discussion With Clinicians About Aspirin Use for Primary Prevention of MI or Stroke: 2015-2020^a^

Variable	No./No. (%)			*P* value, baseline vs year 4
	Baseline	Year 2	Year 4	
**Minnesota**				
Patient initiated	180/1001 (18)	168/896 (19)	193/930 (21)	.12
Clinician initiated	241/1001 (24)	228/896 (26)	240/930 (26)	.38
Any discussion^b^	341/1001 (34)	298/896 (33)	339/930 (36)	.27
**Control states**				
Patient initiated	71/492 (14)	79/456 (17)	76/466 (16)	.42
Clinician initiated	97/492 (20)	109/456 (24)	106/466 (23)	.25
Any discussion^b^	139/492 (28)	146/456 (32)	153/466 (33)	.12

Abbreviation: MI, myocardial infarction.

^a^ Survey questions related to these results (QF1 and QF2) are given in the eAppendix in the Supplement.

^b^ Discussions were patient initiated, clinician initiated, or both.

In Table 4, aspirin use for primary prevention of CVD in Minnesota and the control states is stratified by all use, appropriate use, overuse, underuse, and appropriate nonuse according to the 2009 USPSTF guidelines.^12^ Aspirin use in Minnesota exceeded that in the control states in all surveys. There were modest increases in regular aspirin use between baseline and year 2 surveys with large deceases in the year 4 survey (baseline: 816 of 1974 [41%]; year 4: 629 of 1842 [34%]; *P* < .001). Appropriate aspirin use, as indicated in the guidelines, was stable between baseline and year 2 but decreased in the year 4 survey (year 2: 597 of 1208 [49%]; year 4: 478 of 1191 [40%]; *P* < .001). Appropriate nonuse increased modestly in Minnesota but varied in the other states. Overuse (ie, use of aspirin by individuals without an indication) decreased in Minnesota in the final year but varied in the adjacent states (year 2: 170 of 602 [28%]; year 4: 151 of 651 [23%]; *P* = .04). Underuse (ie, no use of aspirin by individuals with an indication) increased in Minnesota while remaining stable in the adjacent states (Table 4). This increase was more marked in Minnesota, but the differences with the control states were modest.

**Table 4.** Aspirin Use for Primary Prevention of MI or Stroke in Minnesota and Surrounding States: 2015-2020

Variable^a^	Minnesota, No./No. (%)			*P* value, year 2 vs 4	Surrounding states, No./No. (%)			*P* value	
	Baseline	Year 2	Year 4		Baseline	Year 2	Year 4	Year 2 vs 4	Minnesota vs control^b^
All regular aspirin use	816/1974 (41)	767/1810 (42)	629/1842 (34)	<.001	322/973 (33)	304/818 (37)	291/925 (31)	.01	.46
Appropriate indicated use	625/1271 (49)	597/1208 (49)	478/1191 (40)	<.001	241/603 (40)	219/529 (41)	219/591 (37)	.11	.22
Appropriate nonuse	512/703 (73)	432/602 (72)	500/651 (77)	.04	289/370 (78)	204/289 (71)	262/334 (78)	.03	.54
Inappropriate use (overuse)	191/703 (27)	170/602 (28)	151/651 (23)	.04	81/370 (22)	85/289 (29)	72/334 (22)	.03	.54
Indicated not used (underuse)	646/1271 (51)	611/1208 (51)	713/1191 (60)	<.001	362/603 (60)	310/529 (59)	372/591 (63)	.11	.22

Abbreviation: MI, myocardial infarction.

^a^ According to 2009 US Preventive Services Task Force (USPSTF) guidelines, regular aspirin use was defined as every day or every other day to prevent a myocardial infarction or stroke. Aspirin indicated was defined as no self-reported contraindications (gastrointestinal bleed/peptic ulcer, aspirin allergy, or anticoagulant use) and a cardiovascular disease 10-year risk level at or above the USPSTF cutoff. Appropriate indicated use was defined as aspirin was indicated and used regularly. Inappropriate use (overuse) was defined as regular aspirin use when aspirin was not indicated. Indicated not used (underuse) was defined as aspirin indicated but no regular use. Appropriate nonuse was defined as no aspirin use and not indicated.

^b^ *P* values are for the difference in Minnesota vs the difference in control states from year 2 to year 4.

## Discussion

Public health campaigns today have used many strategies. Use of analog broadcast media and print have been mainstays. Clinic approaches through the education of health care professionals and development of screening and treatment protocols are important elements in developing preventive practices at the clinic level. More recently, social media and other web-based digital strategies are increasing in use and exploration.^9,10,29,30^

Although this project used contemporary strategies, the study did not attain the hypothesized goals. Outcome trends in the intervention areas were similar to the controls. Overall use of aspirin for primary CVD prevention decreased between the baseline and last (year 4) surveys. This decrease in use was noted in all individuals who were using aspirin.

The exposures to the intervention program were substantial, particularly in the use of the website. Nonetheless, the numbers of participants reporting hearing or seeing the Ask About Aspirin campaign were modest and decreased at the last survey. It was also apparent, as reported in Table 2, that the number of unfavorable messages about aspirin use significantly increased during the final 2 years of the study. In addition, the number of patient- or physician-initiated discussions, although increasing slightly, did not differ much from those in the control areas.

There are several reasons speculated for these study outcomes. The first relates to the magnitude of intervention seeking to influence the behaviors of more than 1 million adults statewide and thousands of clinicians. More than 100 clinics were recruited and approximately 1000 clinicians completed the continuing medical education program. However, many clinics and clinicians were not directly exposed either through a lack of interest or inadequate resources to provide the education program. Both the length and magnitude of this portion of the intervention may not have been adequate to produce lasting change in busy primary care practices.

There was also considerable optimism about the digital media approach with large numbers of people accessing the website. However, producing widespread and substantial behavior change through social media is still a new and challenging area. Although the website had many hits and thousands of individuals were engaged, we do not know whether they asked their clinicians about aspirin. Other trials have been challenged to note a change in health through digital media.^29,30^ There is still much to learn about using this media in population-wide health programs.

An unexpected finding is the decrease in aspirin use for primary prevention of CVD observed in the year 4 survey (2019-2020). There are several potential explanations. Although clinician adoption of new aspirin guidelines is shown to be slow, the 2016 change in USPSTF recommendations may have had an impact.^31^ These new guidelines downgraded scientific certainty about aspirin use and narrowed the age window recommendation to 50 to 69 years.^13^ We have analyzed our data using the 2016 guidelines but found no differences in the conclusions.

Outcomes may have also been influenced by controversy about aspirin use in the national media during the project. New clinical trials, presented in the fall of 2018, raised questions about aspirin use for primary CVD prevention^32,33,34,35^ as described in a review by Raber et al.^36^

The findings of these trials achieved considerable global media attention and circulated widely in the Upper Midwest. Google search (low-dose aspirin benefits) showed more than 7000 individual news stories appearing in analog and digital media between 2016 and 2020, with many repeated in multiple US media outlets. News stories characterizing aspirin use as risky and dangerous challenged the standard beliefs about aspirin use for prevention of CVD.^37^

### Limitations

This study has limitations. Although the intervention was widespread and intense, most eligible citizens likely remained unexposed.

The use of landline telephones may have introduced bias because individuals with only cell phones were excluded. National surveys find that landlines are more likely to be used by older adults and in more rural areas, such as those we surveyed.^38^ The participation rate of 49%, although not ideal, is similar to that found in successful national surveys.^39^

There is also the potential for spillover effect in the surrounding states. Interstate communication in border areas is common and many of the participating health systems owned clinics in adjacent states. Similarly, the internet is ubiquitous and the study information may have been accessed by individuals in the surrounding control states.

This study used the 2009 US Preventive Services Task Force guidelines. The data were analyzed using the newer 2016 guidelines as well. There were no significant differences in observed outcomes. A new USPSTF report is anticipated in 2022.

## Conclusions

In this nonrandomized controlled trial, a 4-year multichannel campaign using professional education, clinic interventions, and analog and digital media did not appear to produce a populationwide improvement in appropriate aspirin use. Large contextual changes at the national level may have played a role in these negative findings.

## Notes

Supplement 1.
Trial Protocol

Supplement 2. **eAppendix.** Survey for Minnesota and for Surrounding States **eTable 1.** Primary Prevention Survey Participants: Minnesota and Surrounding States **eTable 2.** Characteristics of Primary Prevention Survey Participants: Minnesota and Surrounding States **eFigure 1.** Aspirin Use for the Primary Prevention of Cardiovascular Disease: US Preventive Services Task Force (USPSTF) Recommendation Statement 2009 **eFigure 2.** Aspirin Use for the Primary Prevention of Cardiovascular Disease and Colorectal Cancer: US Preventive Services Task Force (USPSTF) Recommendation Statement 2016 **eFigure 3.** Billboards

Supplement 3.
Data Sharing Statement
