# Supplementary material for: Association of a Community Population and Clinic Education Intervention Program With Guideline-Based Aspirin Use for Primary Prevention of Cardiovascular Disease: A Nonrandomized Controlled Trial
Source: JAMA Netw Open. 2022 May 10;5(5):e2211107. doi: 10.1001/jamanetworkopen.2022.11107 (PMC9092209; doi:10.1001/jamanetworkopen.2022.11107)
Supplement: Supplement 1. — Trial Protocol [file jamanetwopen-e2211107-s001.pdf]

PROTOCOL COVER PAGE

**Protocol Title**	Partners in Prevention - Reducing Heart Attack and Stroke in Minnesota
**Principal Investigator/Faculty Advisor**	Name: Russell V. Luepker, MD
	Department: Cardiovascular Division
	Telephone Number: 612-626-8586
	Email Address: luepk001@umn.edu
**Student Investigator**	Name:
	Current Academic Status (Student, Fellow, Resident):
	Department:
	Telephone Number:
	Institutional Email Address:
**Scientific Assessment**	Nationally-based, federal funding organizations
**IND/IDE # (if applicable)**	N/A
**IND/IDE Holder**	N/A
**Investigational Drug Services # (if applicable)**	N/A
**Version Number/Date:**	V1/07.09.2018

REVISION HISTORY

Revision #	Version Date	Summary of Changes	Consent Change?

Table of Contents

1.0 Objectives 5
2.0 Background 5
3.0 Study Endpoints/Events/Outcomes 5
4.0 Study Intervention(s)/Investigational Agent(s) 6
5.0 Procedures Involved 6
6.0 Data and Specimen Banking 7
7.0 Sharing of Results with Participants 7
8.0 Study Population 7
9.0 Vulnerable Populations 8
10.0 Local Number of Participants 9
11.0 Local Recruitment Methods 9
12.0 Withdrawal of Participants 9
13.0 Risks to Participants 10
14.0 Potential Benefits to Participants 10
15.0 Statistical Considerations 10
16.0 Confidentiality 10
17.0 Provisions to Monitor the Data to Ensure the Safety of Participants 11
18.0 Provisions to Protect the Privacy Interests of Participants 12
19.0 Compensation for Research-Related Injury 12
20.0 Consent Process 12
21.0 Setting 13
22.0 Multi-Site Research 13
23.0 Resources Available 13
24.0 References 14

ABBREVIATIONS/DEFINITIONS

1.0 Objectives

1.1 Purpose: This project will evaluate the use of a health systems intervention, against a background of a mass media campaign, designed to improve the appropriate use of aspirin for the prevention of heart attack and stroke in a high-risk adult population. Using the population of the state of Minnesota as a base, it will develop methods to improve aspirin use at the community level.

2.0 Background

2.1 Significance of Research Question/Purpose: Although significant progress in the reduction of acute myocardial infarction and stroke is apparent, these cardiovascular disorders continue as the leading causes of morbidity and mortality. In recent years, and in the context of the positive results from large randomized clinical trials, there is growing consensus that aspirin, when appropriately used, reduces cardiovascular morbidity in men and women. While use of aspirin to prevent a first heart attack or stroke is beneficial, only about 30-40% of the eligible Minnesota population uses aspirin for the primary prevention of heart attack or stroke. In contrast, 75-85% of individuals who should use aspirin for secondary prevention do so.

2.2 Preliminary Data: In 2012, a pilot study was initiated to test the intervention components and evaluation procedures in the community of Hibbing, MN. Baseline, 4-month and 18-month follow-up telephone surveys of randomly selected adults (men aged 45-79 and women 55-79) resulted in participation rates of 60%. From baseline to follow-up, aspirin use for primary prevention increased significantly from 37% to 52%.

2.3 Existing Literature: Seven major prospective randomized placebo controlled clinical trials over 25 years have examined the efficacy and risk of aspirin use to prevent a first heart attack or stroke. In the United States, the Physicians Health Study and Women’s Health Study evaluated over 60,000 individuals. The 2016 U.S. Preventive Services Task Force (USPSTF) recommendation endorses the use of aspirin for the primary prevention of hearts attacks and strokes in men and women ages 50-59 with a 10% or greater 10-year CVD risk (B recommendation) and men and women ages 60-69 with a 10% or greater 10-year CVD risk (C recommendation).

3.0 Study Endpoints/Events/Outcomes

3.1 Primary Endpoint/Event/Outcome: Self-reported aspirin use for CVD primary prevention in random samples of Minnesota and Upper Midwest men ages 45-79 and women ages 55-79.

3.2 Secondary Endpoint(s)/Event(s)/Outcome(s): N/A

4.0 Study Intervention(s)/Investigational Agent(s)

4.1 Description: The study intervention consists of two educational components: one aimed at increasing public awareness of effective heart attack and stroke prevention, through a variety of media and public relations outlets; and, a second aimed at integrating USPSTF recommendations as a part of a health system's quality improvement (QI) initiative, and providing the education and tools to facilitate the appropriate recommendation of primary prevention aspirin use to patients. The desired action on the part of the public is for men and women in the targeted age groups to consult their health professional(s) to ask if they should take aspirin. The action desired on the part of health professionals is for them to evaluate their patients immediately about the use of aspirin for cardiovascular disease prevention and to recommend it to those individuals for whom it is appropriate.

4.2 Drug/Device Handling: N/A

4.3 Biosafety: N/A

4.4 Stem Cells: N/A

5.0 Procedures Involved

5.1 Study Design: The study design consists of a group-randomized trial (GRT) to evaluate two interventions, a media communications campaign and a health system approach to enhance the effectiveness of care providers to improve appropriate aspirin use among men and women per the USPSTF guidelines. GRTs are characterized by the random assignment of identifiable units rather than individuals to study conditions, with measurements taken on the members of those groups to assess the impact of the intervention. In this study, the groups are 24 defined geographic areas in the State of Minnesota. The independent effects of the media intervention and the joint effects of media plus a health system approach will be evaluated. At baseline, all 24 geographic areas will receive the media communication campaign and only 12 geographic areas will receive the health system intervention. After 2 years, the remaining 12 geographic areas will receive the health system intervention with the media campaign continuing across all 24 areas. To control for secular trends of aspirin use, 4 surrounding states will be evaluated (Iowa, North Dakota, South Dakota and Wisconsin).

5.2 Study Procedures: The primary endpoint of this study, appropriate aspirin use for primary CVD prevention, will be collected by telephone survey in randomly selected individuals ages 45-79. The group-randomized design includes 100 participants in each of 24 selected geographic areas for each survey. 1200 participants will be surveyed across the 4 surrounding states. In addition to verbal consent, the survey will contain demographic questions, questions on aspirin use, CVD status, physician contacts, risk factors, media exposure and other items relevant to understanding program effects. The telephone survey has been administered at baseline (year 2015) and at year 3 (2017). It will also be administered in year 5 (2019).

To assure minority representation in the surveys, 800 in-person surveys of age appropriate individuals from minority groups were surveyed respectively at baseline (year 2015) and at year 3 (2017). The in-person survey of 800 age appropriate individuals from minority groups will also be surveyed in year 5 (2019).

5.3 Study Duration: Individual participants spend approximately 10 minutes answering the questions in the telephone survey and the in-person survey.

- The duration anticipated to enroll all study participants is through 2020.
- The duration anticipated to complete all study procedures, including data analysis is 2021.

5.4 Individually Identifiable Health Information: N/A

5.5 Use of radiation*:* N/A

5.6 Use of Center for Magnetic Resonance Research*:* N/A

6.0 **Data and Specimen Banking** N/A

7.0 Sharing of Results with Participants N/A

8.0 Study Population

8.1 Inclusion Criteria: Men and women ages 45-79 years living in Minnesota, Iowa, North Dakota, South Dakota, and Wisconsin. Men and women ages 45-79 from minority groups living in Minnesota.

8.2 Exclusion Criteria: N/A

8.3 Screening: Individuals will be assessed for eligibility by asking the individual for the year they were born and for their state of residence.

9.0 Vulnerable Populations

9.1 Vulnerable Populations:

- ☐ Children
- ☐ Pregnant women/Fetuses/Neonates
- ☐ Prisoners
- ☐ Adults lacking capacity to consent and/or adults with diminished capacity to consent, including, but not limited to, those with acute medical conditions, psychiatric disorders, neurologic disorders, developmental disorders, and behavioral disorders
- ☐ Approached for participation in research during a stressful situation such as emergency room setting, childbirth (labor), etc.
- ☐ Disadvantaged in the distribution of social goods and services such as income, housing, or healthcare
- ☐ Serious health condition for which there are no satisfactory standard treatments
- ☐ Fear of negative consequences for not participating in the research (e.g. institutionalization, deportation, disclosure of stigmatizing behavior)
- ☐ Any other circumstance/dynamic that could increase vulnerability to coercion or exploitation that might influence consent to research or decision to continue in research
- ☐ Undervalued or disenfranchised social group
- ☐ Members of the military
- X Non-English speakers
- ☐ Those unable to read (illiterate)
- ☐ Employees of the researcher
- ☐ Students of the researcher
- ☐ None of the above

9.2 Additional Safeguards: As part of the NIH guidelines for NIH-funded grants, minority populations need to be represented in the study research. In this study, we have translated the in-person survey into Spanish to allow for the recruitment of predominantly Spanish-speaking individuals. The interviewers are also Spanish-speakers and have been appropriately trained to gather information from these individuals. The survey questions are minimally invasive and are believe to pose low risk to this community.

10.0 Local Number of Participants

10.1 Local Number of Participants to be Consented:

The number of subjects approved to be enrolled for this study is 14040.

11.0 Local Recruitment Methods

11.1 Recruitment Process: The UMN Minnesota Center for Survey Research (MCSR) will mail a pre-telephone survey cover letter to random households across the 24 geographic areas in Minnesota and across the 4 adjacent States, who have been identified as potentially having a man or woman between the ages of 45 and 79. This pre-telephone survey letter will introduce the study and inform individuals that they will be receiving a phone call to conduct a 5-10 minute one-time survey. A week later, MCSR will call these households and screen the recipients for their age-eligibility and interest in participating in the telephone survey.

A community-based organization serving minority populations conducts the in-person interviews at community events or other locations serving these populations.

11.2 Identification of Potential Participants: Publically available lists of landline households are used to contact potential participants for the telephone surveys. For the in person surveys, tables are set up at community events with flyers that indicate the age specifications for the study.

11.3 Recruitment Materials: A pre-survey letter is used in advance of the telephone survey to inform potential participants of the survey call. Flyers at community events are used to recruit individuals from minority groups at community events.

11.4 Payment: No payment is provided to telephone participants. A $10 gift card is provided to individuals from minority groups participating in the in-person interviews.

12.0 Withdrawal of Participants

12.1 Withdrawal Circumstances: N/A

12.2 Withdrawal Procedures: N/A

12.3 Termination Procedures: N/A

13.0 Risks to Participants

13.1 Foreseeable Risks: The 10 minute participation in a one-time telephone survey or in-person survey of CVD health questions and aspirin use is believed to pose minimal risk.

13.2 Reproduction Risks: N/A

13.3 Risks to Others: N/A

14.0 Potential Benefits to Participants

14.1 Potential Benefits: N/A

15.0 Statistical Considerations

15.1 Data Analysis Plan: The design is a member-cross-section Group Randomized Trial as respondents at baseline and at follow-up will not be restricted to be the same persons. We will use a mixed-model logistic regression analysis of 2 and 4-year follow-up aspirin use data, adjusting for baseline mean levels and covariates.

15.2 Power Analysis: For 100 members per geographic area, a two-tailed Type 1 error rate of 5% will have 85% power for an absolute intervention effect of 8/1%, e.g., 30% vs. 38.1%; at 80% power the detectable difference would be 7.6%.

15.3 Statistical Analysis: The mixed-model logistic regression analyses will be fit using SAS PROC GLIMMIX specifying a logit link and a binomial error. We will assess the null hypothesis of no joint intervention effect by testing the randomization condition (health system vs. media comparison) against the area-level (cluster) error term. Surveys will balance the numbers of men and women so as to allow the most sensitive analyses with gender strata.

15.4 Data Integrity: Trained interviewers are used for both the telephone and in-person surveys.

16.0 Confidentiality

16.1 Data Security: For the telephone surveys, personal identifiers other than zip codes are kept separate from the storage, use, and transmission of data. The University of Minnesota's Center for Survey Research is contracted to collect the data. The data files they send the study researchers include gender, zip code, state of residence, and birth year. For the in-person surveys, no personal identifiers are collected, and only gender, birth year, and state of residence is known. Verbal consent is sought for both.

17.0 Provisions to Monitor the Data to Ensure the Safety of Participants

17.1 Data Integrity Monitoring. Dr. Luepker, PI, monitors data collection efforts on a monthly basis as a data quality check.

17.2 Data Safety Monitoring. This is a minimal risk study with regards to the specific research interaction with telephone and in-person survey participants to collect attitudes, beliefs and behaviors around primary prevention aspirin use.

Since the study's interventions promote the USPSTF guidelines for primary prevention aspirin use on a community-wide basis, we intend to track major bleeding disorders by comprehensive surveillance of all hospitals in the state via the Minnesota Hospital Association. The recommendation of aspirin has the potential adverse effects of gastrointestinal bleeding and hemorrhagic stroke. In this program, we are following national recommendations for appropriate use of aspirin in those groups where it is known that benefits outweigh adverse events. Our media approach recommends discussion with a health professional before staring aspirin use. We note from retrospective data that dramatic increases in aspirin use have not been associated with either increased gastrointestinal bleeding or increased hemorrhagic stroke on the community level.

A DSMB has been established and meets yearly. Their responsibilities include the following:

a. Review the research protocol and plans for data and safety monitoring.
b. Evaluate study progress including periodic assessments of data quality and timeliness, participant recruitment and other factors that may affect the study outcomes.
c. Consider external factors to the study when relevant information becomes available such as scientific or therapeutic developments that may affect the study progress.

d. Protect the safety of the study participants.

e. Report on the progress of the study.

f. Make recommendations to the NHLBI, the grantee institution, the principal investigators and the Institutional Review Board concerning continuation, termination or other modifications of the study.

g. Review survey data for quality and consistency considering the proposed outcomes postulated for the study.

h. Assist the study investigators by commenting on any problems with study conduct, enrollment and/or data collection.

18.0 Provisions to Protect the Privacy Interests of Participants

18.1 Protecting Privacy: Both the telephone and in-person interviews are voluntary. Privacy is maintained during the telephone interviews since it is conducted in the privacy of the participant's home. In-person interviews are conducted at tables on the side-lines of community events. The 10 minute survey will occur in a relaxed and open atmosphere conducive for private conversation.

18.2 Access to Participants*:* All information gathered from participants is via self-reports from the telephone or in-person interviews. Medical records or any other sources of private information are not being accessed.

19.0 Compensation for Research-Related Injury

19.1 Compensation for Research-Related Injury: N/A

19.2 Contract Language: N/A

20.0 Consent Process

20.1 Consent Process (when consent will be obtained): Verbal consent is maintained at the time of the telephone or in-person interview. Telephone participants are provided the opportunity to learn about the study in advance of the telephone survey via a pre-survey letter mailed to the household. In-person survey participants are able to see the study recruitment materials prior to approaching the interviewers and/or they are informed about the study prior to beginning the actual survey.

20.2 Waiver or Alteration of Consent Process (when consent will not be obtained): N/A

20.3 Non-English Speaking Participants: To accommodate Spanish speakers whose primary language is Spanish, the in-person surveys have been translated into Spanish and Spanish-speaking trained interviewers conduct the interviews.

20.4 Participants Who Are Not Yet Adults (infants, children, teenagers under 18 years of age): N/A

20.5 Cognitively Impaired Adults, or adults with fluctuating or diminished capacity to consent: N/A

20.6 Adults Unable to Consent: N/A

- Permission:
- Assent:
- Dissent:

21.0 Setting

21.1 Research Sites: Telephone interviews are conducted across the State of Minnesota, including 4 adjacent states (Iowa, North Dakota, South Dakota, Wisconsin). The media intervention uses a variety of media channels across the State of Minnesota. The health system intervention is engaging health systems and family practice clinics across the State of Minnesota.

21.2 International Research: N/A

22.0 **Multi-Site Research** N/A

23.0 Resources Available

23.1 Resources Available: The University of Minnesota's Minnesota Heart Health Program, which has existed for 30+ years, has the appropriate resources necessary to conduct this study.

24.0 References
