# Supplementary material for: Association of a Community Population and Clinic Education Intervention Program With Guideline-Based Aspirin Use for Primary Prevention of Cardiovascular Disease: A Nonrandomized Controlled Trial
Source: JAMA Netw Open. 2022 May 10;5(5):e2211107. doi: 10.1001/jamanetworkopen.2022.11107 (PMC9092209; doi:10.1001/jamanetworkopen.2022.11107)
Supplement: Supplement 2. — eAppendix. Survey for Minnesota and for Surrounding States eTable 1. Primary Prevention Survey Participants: Minnesota and Surrounding States eTable 2. Characteristics of Primary Prevention Survey Participants: Minnesota and Surrounding States eFigure 1. Aspirin Use for the Primary Prevention of Cardiovascular Disease: US Preventive Services Task Force (USPSTF) Recommendation Statement 2009 eFigure 2. Aspirin Use for the Primary Prevention of Cardiovascular Disease and Colorectal Cancer: US Preventive Services Task Force (USPSTF) Recommendation Statement 2016 eFigure 3. Billboards [file jamanetwopen-e2211107-s002.pdf]

Supplementary Online Content

Luepker RV, Eder M, Finnegan JR, Van’t Hof JR, Oldenburg N, Duval S. Association of a community population and clinic education intervention program with guideline-based aspirin use for primary prevention of cardiovascular disease: a nonrandomized controlled trial. *JAMA Netw Open*. 2022;5(5):e2211107. doi:10.1001/jamanetworkopen.2022.11107

eAppendix. Survey for Minnesota and for Surrounding States

eTable 1. Primary Prevention Survey Participants: Minnesota and Surrounding States

eTable 2. Characteristics of Primary Prevention Survey Participants: Minnesota and Surrounding States

eFigure 1. Aspirin Use for the Primary Prevention of Cardiovascular Disease: US Preventive Services Task Force (USPSTF) Recommendation Statement 2009

eFigure 2. Aspirin Use for the Primary Prevention of Cardiovascular Disease and Colorectal Cancer: US Preventive Services Task Force (USPSTF) Recommendation Statement 2016

eFigure 3. Billboards

This supplementary material has been provided by the authors to give readers additional information about their work.

© 2022 Luepker RV et al. *JAMA Network Open.*

eAppendix. Survey for Minnesota and for Surrounding States

Section A
Introduction and Qualifying Questions

INTRODUCTION: "Hello, my name is _(name)_______ . I'm calling on behalf of the University of Minnesota and the health care providers in your local area. We are conducting a survey about the prevention of heart attack and stroke, so that we can develop programs to help people live healthier lives. A letter was recently sent to your household about this survey.

QA1. Your household was chosen from a random sample of households in your area. We're interested in talking to men and women in specific age groups.

Is there a male between 45 and 79 years old living in your household?

1. Yes, speaking ➔ ***SKIP TO SECTION B***
2. Yes, someone else ➔ ***IF YES, someone else:*** **May I speak with him?**

    IF YES: repeat Introduction, then SKIP TO QB2
    IF NO: " **Is there another time I could call back to talk to that person?"**

    IF YES: take call back information
    IF NO: **GO TO QA2**

0. No
8. DK
9. RA

QA2. Is there a female between 55 and 79 years old living in your household?

1. Yes, speaking ➔ ***SKIP TO SECTION B***
2. Yes, someone else ➔ ***IF YES, someone else:*** **"May I speak with her?"**

    IF YES: repeat Introduction, then SKIP TO QB2
    IF NO: "**Is there another time I could call back to talk to that person?"**

    IF YES: take call back information
    IF NO: **"Thanks for your time. Good bye."**

0. No
8. DK
9. RA
. NA

© 2022 Luepker RV et al. *JAMA Network Open.*

IF NO TO BOTH QA1 AND QA2: **"Since there are no men or women in our specific age groups in your household those are all the questions that I have for you. Thanks very much for your time. Good bye."**

Section B
Confirmation of Age and Location

QB1. (DO NOT ASK) Is respondent male or female?

1. Male (IF RESPONDENT SAID YES TO QA1)
0. Female (IF RESPONDENT SAID YES TO QA2)

QB2. First, to confirm that you are eligible to complete the survey, what year were you born?

____ ____ ____ ____ ***(FILL IN 4 DIGIT YEAR)***

(MEN MUST BE BORN BETWEEN 1936 AND 1970 TO CONTINUE)

(WOMEN MUST BE BORN BETWEEN 1936 AND 1960 TO CONTINUE)

IF NOT AGE ELIGIBLE: "Since you are not in the specific age group that we are looking for, those are all the questions that I have for you. Thank you for your time. Good bye."

QB3. What is the state where your main residence is located?

1. Minnesota
2. Iowa
3. North Dakota
4. South Dakota
5. Wisconsin
6. Other (THANK AND TERMINATE)
8. DK (THANK AND TERMINATE)
9. RA (THANK AND TERMINATE)

"Since your main residence is not in any of the states that we are looking for, those are all the questions that I have for you. Thank you for your time. Good bye."

QB4. What is the zipcode of your main residence?

____ ____ ____ ____
88888. DK
99999. RA

© 2022 Luepker RV et al. *JAMA Network Open.*

QB4a. (IF DK OR RA) What city is the address for your main residence?

____

IF NOT ZIPCODE ELIGIBLE: "Since your main residence is not in any of the zip codes that we are looking for, those are all the questions that I have for you. Thank you for your time. Good bye."

Section C
Verbal Consent

The survey is completely voluntary. You may refuse to answer any question or stop at any time. All of your responses to the survey will be kept confidential and you will never be identified in any reports.

Section D
Cardiovascular Health

Now I have a few questions about your heart health.

QD1. Have you ever been told by a doctor or other health professional that you had high blood pressure?

1. Yes
0. No
8. DK
9. RA

QD2. Have you ever been told by a doctor or other health professional that you had high blood cholesterol?

1. Yes
0. No
8. DK
9. RA

© 2022 Luepker RV et al. *JAMA Network Open.*

QD3. Have you ever been told by a doctor or other health professional that you had diabetes?

(INTERVIEWER: THIS DOES **NOT** INCLUDE GESTATIONAL DIABETES**)**

1. Yes
0. No
8. DK
9. RA

QD4. Have you ever been told by a doctor or other health professional (READ LIST)?

	YES	NO	DK	RA
**QD4a. that you had a heart attack**	1	0	8	9
**QD4b. that you had a stroke**	1	0	8	9
**QD4c. that you had peripheral artery disease or blockages in your leg arteries or decreased blood flow to your legs**	1	0	8	9

QD5. Have you ever had a procedure to open up or bypass blocked arteries in your heart, leg, or neck?

(INTERVIEWER: THIS DOES **NOT** INCLUDE DIAGNOSTIC ANGIOGRAMS**)**

1. Yes
0. No
8. DK
9. RA

QD6. Have you ever been told by a doctor or other health professional (READ LIST)?

	YES	NO	DK	RA
**QD6a. that you had an ulcer or bleeding in your stomach or intestines**	**1**	**0**	**8**	**9**
**QD6b. that you are allergic to aspirin**	**1**	**0**	**8**	**9**

QD7. Do you regularly take prescription blood thinners or any anti-clotting or clot-preventing drugs, such as warfarin or Coumadin, clopidogrel or Plavix, or others?

© 2022 Luepker RV et al. *JAMA Network Open.*

1. Yes
0. No
8. DK
9. RA

QD8. Have you ever smoked cigarettes on a regular basis, that is, more than 100 cigarettes in your lifetime?

1. Yes
0. No
8. DK
9. RA

QD9. Do you smoke at present?

1. Yes
0. No
8. DK
9. RA

Section E
Aspirin Use

QE1. Do you take aspirin to prevent a heart attack or stroke?

1. Yes SKIP TO QE2
0. No
8. DK
9. RA

QE1a. (IF NO, DK, OR RA) Have you taken aspirin in the past to prevent heart attack or stroke, and then stopped?

1. Yes
0. No SKIP TO SECTION F
8. DK SKIP TO SECTION F
9. RA SKIP TO SECTION F
. NA

QE1a-1. (IF YES) Why did you STOP taking aspirin to prevent heart attack or stroke? (INTERVIEWER: DO NOT READ LIST; SELECT ALL THAT APPLY)

© 2022 Luepker RV et al. *JAMA Network Open.*

a. Bruising
b. Stomach or intestinal bleeding
c. Health professional told me to stop
d. Aspirin allergy/other allergies
e. Started a new medicine
f. Dyspepsia or heart burn
g. Don't think it works
h. Heard/read/saw something in the news that frightened me
i. Aspirin costs too much
j. Don't like taking medication/too many pills
k. Don't think I am at risk
l. Other (specify) ________________________________
m. DK
n. RA
. NA

(IF NO, DK, OR RA TO QE1, SKIP TO SECTION F)

QE2. How often do you take aspirin to prevent a heart attack or stroke? (DO NOT READ LIST UNLESS NEEDED)

1. Daily
2. Every other day
3. Once a week or less often
8. DK
9. RA
. NA

QE3. How many years have you been taking aspirin to prevent a heart attack or stroke?

1. Less than 1 year	
2. 1 to 2 years	SKIP TO SECTION F
3. More than 2 years	SKIP TO SECTION F
8. DK	SKIP TO SECTION F
9. RA	SKIP TO SECTION F
. NA	

QE3a. (IF LESS THAN ONE YEAR) How many months?

_____ ____ **# OF MONTHS**

88. DK
99. RA
. NA

© 2022 Luepker RV et al. *JAMA Network Open.*

Section F
Interventions: Health System and Mass Media

Now I have a few questions about the sources you may use to make decisions about your health.

I will first ask you about the doctors or other health professionals that you may see.

QF1. In the past 2 years, did you ASK a doctor or other health professional whether you should use aspirin as a means to prevent a heart attack or stroke?

1. Yes
0. No
8. DK
9. RA

QF2. In the past 2 years, has a doctor or health professional STARTED A DISCUSSION with you about whether you should use aspirin to prevent a heart attack or stroke?

1. Yes
0. No
8. DK
9. RA

QF3. In the past 2 years, have you had a regular provider of health care?

1. Yes
0. No
8. DK
9. RA

QF4. In the past 2 years, have you seen or heard anything (READ LIST) about taking aspirin to prevent a heart attack or stroke?

		YES	NO	DK	RA
**QF4a.**	**In your community**	1	0	8	9
**QF4b.**	**At your workplace**	1	0	8	9
**QF4c.**	**Where you receive health care**	1	0	8	9

© 2022 Luepker RV et al. *JAMA Network Open.*

(IF NO, DK, or RA TO QF4a AND QF4b AND QF4c, SKIP TO SECTION G)

QF5. Have the messages you have seen or heard been in FAVOR or NOT in favor of a person using aspirin to prevent a FIRST TIME heart attack or stroke?

1. In FAVOR
0. NOT in favor
8. DK
9. RA
. NA

Section G
Additional Demographic Information

Before ending this survey, I have just a few remaining background questions.

QG1. Are you married, single, divorced, separated, or widowed?

1. Married
2. Single
3. Divorced
4. Separated
5. Widowed
6. Other (specify) ___________________________
8. DK
9. RA

QG2. What is the highest level of school you have completed? (*DO NOT READ LIST; CLARIFY "HIGH SCHOOL" OR "COLLEGE"*)

01. Less than high school
02. Some high school
03. High school graduate
04. Some technical school or 2 year community college
05. Technical school or 2 year community college graduate
06. Attended a 4 year college but did NOT graduate
07. College graduate (Bachelor's degree, BA, BS)
08. Some graduate school or professional school
09. Post graduate or professional degree (Master's, Doctorate, MS, MA, PhD, Law degree, Medical degree)
10. Other (specify) ______________________________
88. DK
99. RA

© 2022 Luepker RV et al. *JAMA Network Open.*

QG3. Are you Spanish, Hispanic, or Latino?

1. Yes
0. No
8. DK
9. RA

QG4. Do you consider yourself to be White, Black or African American, American Indian or Alaska Native, Asian, or Native Hawaiian or Other Pacific Islander? (*Select all that apply)*

1. White
2. Black or African American
3. American Indian or Alaska Native
4. Asian
5. Native Hawaiian or Other Pacific Islander
6. Other (specify) ________________________
8. DK
9. RA

© 2022 Luepker RV et al. *JAMA Network Open.*

eTable 1. Primary Prevention Survey Participants: Minnesota and Surrounding States

Minnesota (n=5,626)

	Baseline		Year 2		Year 4	
**Age group**	**Women n**	**Men n**	**Women n**	**Men n**	**Women n**	**Men n**
45-54	-	207	-	142	-	132
55-64	393	357	328	323	305	296
65-74	420	311	379	353	427	370
75-79	188	98	189	96	198	114
**TOTAL N (%)**	1,001 (51%)	973 (49%)	896 (50%)	914 (50%)	930 (50%)	912 (50%)

Surrounding States (n=2,716)

	Baseline		Year 2		Year 4	
**Age group**	**Women n**	**Men n**	**Women n**	**Men n**	**Women n**	**Men n**
45-54	-	106	-	62	-	77
55-64	209	176	157	120	157	160
65-74	201	158	196	142	219	175
75-79	82	41	103	38	90	47
**TOTAL N (%)**	492 (51%)	481 (49%)	456 (56%)	362 (44%)	466 (50%)	459 (50%)

© 2022 Luepker RV et al. *JAMA Network Open.*

eTable 2. Characteristics of Primary Prevention Survey Participants: Minnesota and Surrounding States

		Minnesota			Surrounding States			
		**Baseline No (%)**	**Year 2 No (%)**	**Year 4 No (%)**	**Baseline No (%)**	**Year 2 No (%)**	**Year 4 No (%)**	***p*-value[a]**
n		1,974	1,810	1,842	973	818	925	
Demographics								
Age, years (median, IQR)		64.7 (64.4, 65.1)	65.6 (65.3, 66.0)	66.2 (65.8, 66.5)	64.3 (63.8, 64.8)	66.0 (65.5, 66.6)	65.7 (65.2, 66.2)	0.83
Race								0.59
	White	1,925 (97.5)	1,756 (97.0)	1,799 (97.7)	937 (96.3)	773 (94.4)	888 (95.8)	
	Other [b]	49 (2.5)	54 (3.0)	43 (2.3)	36 (3.7)	45 (5.6)	37 (4.2)	
Education								0.28
	<High school	65 (3.3)	58 (3.2)	43 (2.3)	25 (2.6)	31 (3.8)	19 (2.1)	
	High school graduate	566 (28.8)	530 (29.5)	478 (26.0)	276 (28.4)	215 (26.6)	271 (29.2)	
	Some college	676 (34.4)	649 (36.1)	675 (36.7)	291 (30.0)	237 (28.9)	270 (29.0)	
	College graduate	657 (33.5)	562 (31.2)	643 (35.0)	378 (39.0)	332 (40.7)	363 (39.7)	
Risk factors								
Hypertension		924 (46.8)	865 (47.8)	896 (48.6)	442 (45.4)	412 (50.4)	446 (48.3)	0.73
Hyperlipidemia		870 (44.1)	811 (44.8)	802 (43.5)	425 (43.7)	355 (43.2)	409 (44.2)	0.71
Diabetes		259 (13.1)	271 (15.0)	295 (16.0)	127 (13.1)	134 (16.3)	148 (15.9)	0.94
Current smoker		215 (10.9)	182 (10.1)	198 (10.8)	100 (10.2)	78 (9.5)	93 (9.9)	0.91
Smoking status								0.21
	Never	1,091 (55.3)	1,017 (56.2)	1,057 (57.4)	545 (56.1)	491 (60.2)	575 (62.0)	
	Former	668 (33.8)	611 (33.8)	587 (31.9)	328 (33.7)	249 (30.3)	257 (28.1)	
	Current	215 (10.9)	182 (10.1)	198 (10.8)	100 (10.2)	78 (9.5)	93 (9.9)	
Number of risk factors								0.71
	None	573 (29.0)	526 (29.1)	511 (27.7)	299 (30.8)	231 (28.4)	258 (28.0)	
	1	730 (37.0)	619 (34.2)	671 (36.4)	342 (35.1)	286 (34.8)	334 (36.1)	
	2	484 (24.5)	495 (27.4)	473 (25.7)	249 (25.6)	218 (26.6)	240 (25.8)	
	3	178 (9.0)	160 (8.8)	174 (9.5)	78 (8.0)	75 (9.1)	90 (9.7)	

© 2022 Luepker RV et al. *JAMA Network Open.*

	4	9 (0.5)	10 (0.6)	13 (0.7)	5 (0.5)	8 (1.0)	3 (0.3)	
		**Minnesota**			**Surrounding States**			
		**Baseline**	**Year 2**	**Year 4**	**Baseline**	**Year 2**	**Year 4**	***p*-value[b]**
Aspirin contraindications								
GI bleed/peptic ulcer		144 (7.3)	101 (5.6)	148 (8.0)	78 (8.0)	57 (7.0)	67 (7.3)	0.31
Aspirin allergy		39 (2.0)	46 (2.5)	47 (2.6)	22 (2.2)	17 (2.1)	11 (1.2)	0.034
Anticoagulant use		96 (4.9)	107 (5.9)	117 (6.4)	57 (5.9)	54 (6.6)	69 (7.6)	0.99

[a] *p*-value for comparison of trend across surveys between Minnesota and surrounding states. Risk factors are hypertension, diabetes, hyperlipidemia, diabetes.
IQR: interquartile range; GI: gastrointestinal.
[b] Other races across surveys: Minnesota (African American 0.07%, Native American 0.68%, Asian 0.07%, Pacific Islander 0.07%, Multiracial 0.66%, Unknown 1.05%). Surrounding states (African American 1.36%, Native American 0.77%, Asian 0.15%, Pacific Islander 0.07%, Multiracial 0.59%, Unknown 1.4%)

© 2022 Luepker RV et al. *JAMA Network Open.*

eFigure 1. Aspirin Use for the Primary Prevention of Cardiovascular Disease: US Preventive Services Task Force (USPSTF) Recommendation Statement 2009

ASPIRIN FOR THE PREVENTION OF CARDIOVASCULAR DISEASE
CLINICAL SUMMARY OF U.S. PREVENTIVE SERVICES TASK FORCE RECOMMENDATION

Population	Men Age 45–79 Years	Women Age 55–79 Years	Men Age <45 Years	Women Age <55 Years	Men and Women Age ≥80 Years
Recommendation	Encourage aspirin use when potential CVD benefit (MIs prevented) outweighs potential harm of GI hemorrhage	Encourage aspirin use when potential CVD benefit (strokes prevented) outweighs potential harm of GI hemorrhage	Do not encourage aspirin use for MI prevention	Do not encourage aspirin use for stroke prevention	No Recommendation
	Grade: A		Grade: D		Grade: I (insufficient evidence)

How to Use This Recommendation	Shared decision making is strongly encouraged with individuals whose risk is close to (either above or below) the estimates of 10-year risk levels indicated below. As the potential CVD benefit increases above harms, the recommendation to take aspirin should become stronger.<br><br>To determine whether the potential benefit of MIs prevented (men) and strokes prevented (women) outweighs the potential harm of increased GI hemorrhage, both 10-year CVD risk and age must be considered.

Risk Level at Which CVD Events Prevented (Benefit) Exceeds GI Harms

Men		Women	
Age	10-Year CHD Risk	Age	10-Year Stroke Risk
45–59 years	≥4%	55–59 years	≥3%
60–69 years	≥9%	60–69 years	≥8%
70–79 years	≥12%	70–79 years	≥11%

The table above applies to adults who are not taking NSAIDs and who do not have upper GI pain or a history of GI ulcers. NSAID use and history of GI ulcers increase the risk for serious GI bleeding events considerably and should be considered in determining the balance of benefits and harms.

NSAID use combined with aspirin use approximately quadruples the risk for serious GI bleeding events compared with the risk with aspirin use alone. The rate of serious bleeding in aspirin users is approximately 2 to 3 times greater in patients with a history of GI ulcers.

Risk Assessment	**For men:** Risk factors for CHD include age, diabetes, total cholesterol level, HDL cholesterol level, blood pressure, and smoking.<br>**CHD risk estimation tool:** http://healthlink.mcw.edu/article/923521437.html<br><br>**For women:** Risk factors for ischemic stroke include age, high blood pressure, diabetes, smoking, history of CVD, atrial fibrillation, and left ventricular hypertrophy.<br>**Stroke risk estimation tool:** www.westernstroke.org/PersonalStrokeRisk1.xls
Relevant Recommendations from the USPSTF	The USPSTF has made recommendations on screening for abdominal aortic aneurysm, carotid artery stenosis, CHD, high blood pressure, lipid disorders, and peripheral arterial disease. These recommendations are available at www.preventiveservices.ahrq.gov.

For the full recommendation statement and supporting documents, please go to www.preventiveservices.ahrq.gov.

CHD = coronary heart disease; CVD = cardiovascular disease; GI = gastrointestinal; HDL = high-density lipoprotein; MI = myocardial infarction; NSAID = nonsteroidal anti-inflammatory drug; USPSTF = U.S. Preventive Services Task Force.

Reference

Aspirin for the Prevention of Cardiovascular Disease: U.S. Preventive Services Task Force Recommendation Statement. Annals of Internal Medicine 2009;150:396-404. [Epub ahead of print 17 March 2009]. doi:https://doi.org/10.7326/0003-4819-150-6-200903170-00008

© 2022 Luepker RV et al. *JAMA Network Open.*

eFigure 2. Aspirin Use for the Primary Prevention of Cardiovascular Disease and Colorectal Cancer: US Preventive Services Task Force (USPSTF) Recommendation Statement 2016

Population	Adults aged 50 to 59 y with a ≥10% 10-y CVD risk	Adults aged 60 to 69 y with a ≥10% 10-y CVD risk	Adults younger than 50 y	Adults aged 70 y or older
Recommendation	Initiate low-dose aspirin use. Grade: B	The decision to initiate low-dose aspirin use is an individual one. Grade: C	No recommendation. Grade: I (insufficient evidence)	No recommendation. Grade: I (insufficient evidence)

Risk Assessment	Primary risk factors for CVD are older age, male sex, race/ethnicity, abnormal lipid levels, high blood pressure, diabetes, and smoking. Risk factors for GI bleeding with aspirin use include higher aspirin dose and longer duration of use, history of GI ulcers or upper GI pain, bleeding disorders, renal failure, severe liver disease, and thrombocytopenia.<br>The USPSTF used a calculator derived from the ACC/AHA pooled cohort equations to predict 10-y risk for first atherosclerotic CVD event.			
Preventive Medication	Aspirin's anticlotting effect is useful for primary and secondary CVD prevention because it potentially decreases the accumulation of blood clots that form as a result of reduced blood flow at atherosclerotic plaques, thereby reducing hypoxic damage to heart and brain tissue. The mechanisms for inhibition of adenoma or colorectal cancer development are not yet well-understood but may result from aspirin's anti-inflammatory properties.			
Treatment and Dosage	A reasonable approach consistent with the evidence is to prescribe 81 mg/d (the most commonly prescribed dose in the United States), and assess CVD and bleeding risk factors starting at age 50 y and periodically thereafter, as well as when CVD and bleeding risk factors are first detected or change.			
Balance of Benefits and Harms	The benefits of aspirin use outweigh the increased risk for bleeding by a moderate amount.	The benefits of aspirin use outweigh the increased risk for bleeding by a small amount.	The evidence on aspirin use is insufficient and the balance of benefits and harms cannot be determined.	The evidence on aspirin use is insufficient and the balance of benefits and harms cannot be determined.
Other Relevant USPSTF Recommendations	The USPSTF has made recommendations on smoking cessation and promoting a healthful diet and physical activity, as well as screening for carotid artery stenosis, coronary heart disease, high blood pressure, lipid disorders, obesity, diabetes, peripheral artery disease, and colorectal cancer. These recommendations are available on the USPSTF Web site (www.uspreventiveservicestaskforce.org).			

For a summary of the evidence systematically reviewed in making this recommendation, the full recommendation statement, and supporting documents, please go to www.uspreventiveservicestaskforce.org.

Reference
Aspirin Use for the Primary Prevention of Cardiovascular Disease and Colorectal Cancer: U.S. Preventive Services Task Force Recommendation Statement. Annals of Internal Medicine 2016;164:836-845. [Epub ahead of print 12 April 2016]. doi:https://doi.org/10.7326/M16-0577

© 2022 Luepker RV et al. *JAMA Network Open.*

eFigure 3. Billboards

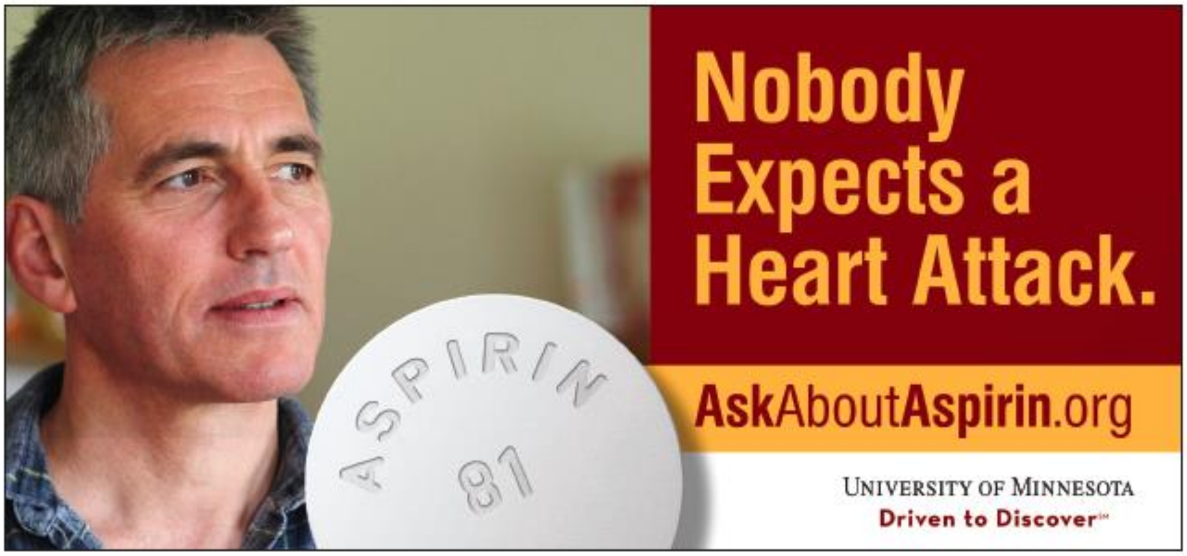

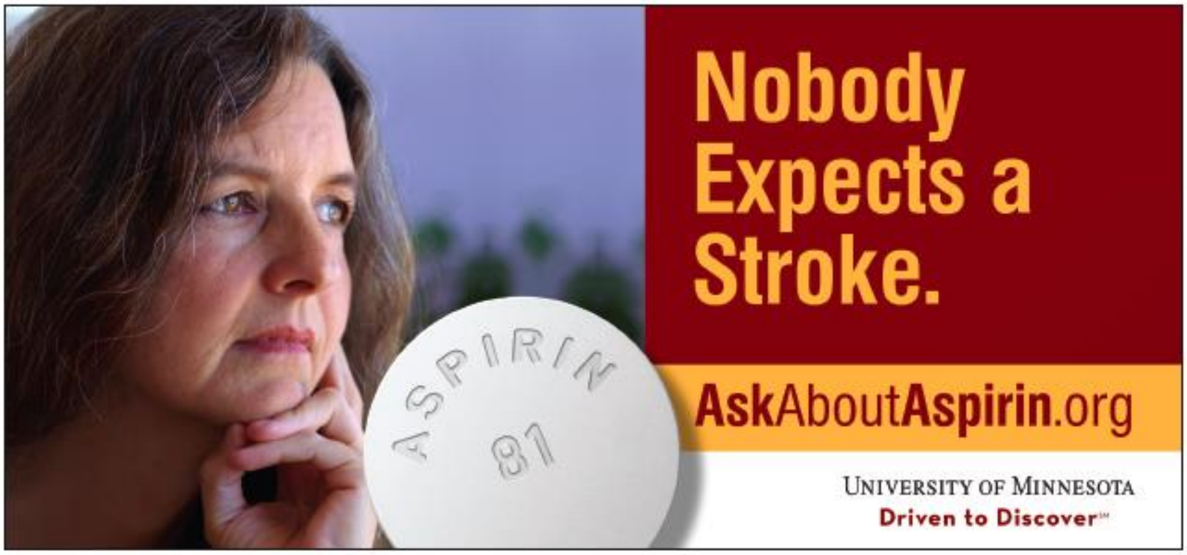

© 2022 Luepker RV et al. *JAMA Network Open.*
