# Supplementary material for: Association of a Community Population and Clinic Education Intervention Program With Guideline-Based Aspirin Use for Primary Prevention of Cardiovascular Disease: A Nonrandomized Controlled Trial
Source: JAMA Netw Open. 2022 May 10;5(5):e2211107. doi: 10.1001/jamanetworkopen.2022.11107 (PMC9092209; doi:10.1001/jamanetworkopen.2022.11107)
Supplement: Supplement 3. — Data Sharing Statement [file jamanetwopen-e2211107-s00.pdf]

Data Sharing Statement

Data
Data available: Yes
How to access data: Corresponding Author Russell V. Luepker, MD, MS. luepk001@umn.edu
When available: With publication
